# *PMS2* mutation spectra in Norway and risk of cancer for carriers of pathogenic variants

**DOI:** 10.1186/s13053-024-00292-6

**Published:** 2024-09-27

**Authors:** Wenche Sjursen, Hanne K. Hyldebrandt, Liss Anne S. Lavik, Bjørn Ivar Haukanes, Sarah Ariansen, Siri Briskemyr, Anna E. Sylvander, Marianne T. Haavind, Maren F. Olsen, Elin S. Røyset, Hildegunn Vetti, Astrid Stormorken, Eli Marie Grindedal

**Affiliations:** 1 Department of Medical Genetics, St Olavs University Hospital, Trondheim, Norway; 2 Department of Clinical and Molecular Medicine, Norwegian University of Science and Technology, Trondheim, Norway; 3 Department of Medical Genetics, Oslo University Hospital, Trondheim, Norway; 4 Institute of Clinical Medicine, University of Oslo, Oslo, Norway; 5 Department of Medical Genetics, Haukeland University Hospital, Trondheim, Norway; 6 Department of Medical Genetics, University Hospital of Northern Norway, Tromsø, Norway; 7 Department of Pathology, St Olavs University Hospital, Trondheim, Norway

**Keywords:** Lync syndrome, PMS2, Cancer risk

## Abstract

**Background:**

In Norway, we have offered testing of *PMS2* since 2006, and have a large national cohort of carriers. The aim of this study was to describe all *PMS2* variants identified, and to describe frequency, spectrum and penetrance of cancers in carriers of class 4/5 variants.

**Methods:**

All detected *PMS2* variants were collected from the diagnostic laboratories and reclassified according to ACMG criteria and gene specific guidelines. Data on variant, gender, cancer diagnosis, age at diagnosis, and age at last known follow-up was collected on all carriers of class 4/5 variants from electronic patient records. The Kaplan-Meier algorithm was used to calculate cumulative risk of any cancer, colorectal cancer and endometrial cancer.

**Results:**

In total, 220 different *PMS2* variants were detected. Twenty nine class 4/5 variants were identified in 482 carriers. The most common pathogenic variant was the founder mutation c.989-1G > T, detected in 204 patients from 58 families. Eighty seven out of 482 (18.0%) had been diagnosed with colorectal cancer, 10 of these (11.8%) before 40 years. Cumulative risk at 70 years in our cohort was 34.7% for colorectal cancer and 26.1% for endometrial cancer.

**Conclusions:**

After 15 years of genetic testing, 29 different class 4/5 variants have been detected in Norway. Almost half of Norwegian *PMS2* carriers have the founder variant 989-1G > T. Penetrance of colorectal cancer in our cohort was moderate but variable, as 11.5% of those diagnosed were younger than 40 years.

**Supplementary Information:**

The online version contains supplementary material available at 10.1186/s13053-024-00292-6.

## Background

Lynch Syndrome (LS) (OMIM #120435) is the most frequent inherited cause of colorectal cancer (CRC) [1]. It is also associated with increased risk of other cancers, such as endometrial, ovarian, prostate, hepatobiliary, urinary, small bowel, brain and sebaceous tumours. LS is caused by pathogenic variants in one of the four mismatch repair (MMR) genes; *MLH1*,* MSH2*,* MSH6* and *PMS2*, and deletions of *EPCAM* leading to aberrant MSH2 protein expression. Penetrance and tumour spectrum vary between the different genes. *MSH2* and *MLH1* are associated with the highest risk of colorectal cancer, while *MSH6* and *PMS2* are less penetrant [2]. Even though cancer risk is different for the different genes, MMR carriers are recommended the same surveillance in Norway; biannual colonoscopies from the age of 25 for early detection and prevention of CRC. Women are offered annual transvaginal ultrasound of the endometrium and ovaries from the age of 30.

The most recent studies indicate that the penetrance of pathogenic variants in *PMS2* is low [2–4]. Some guidelines suggest that *PMS2* carriers should postpone start of colonoscopic surveillance from 25 to 35 years, and that surveillance is done every three to five years instead of every second year [4, 5]. *PMS2* (OMIM * 600259) is nevertheless the least described of the four MMR genes, both in terms of mutation spectra and penetrance of cancer. Due to the lower risk of cancer compared to the other genes, many *PMS2* families likely did not fulfil the original or revised Amsterdam or Bethesda criteria for testing [6], and thus were not identified when these criteria were used to select patients for testing [6, 7]. After genetic testing became more available, and we started to offer multigene panels, we identify these families more frequently.

In Norway, we have performed genetic testing of *PMS2* for more than 15 years. The analyses have included sequencing and Multiplex Ligation-dependent Probe Amplification (MLPA) for copy number detection in DNA, and sequencing of cDNA from selected samples. When tumour tissue was available, immunohistochemistry (IHC) and microsatellite instability (MSI) analyses were performed. The inclusion criteria for testing have changed over the years and have not been limited to only those fulfilling strict clinical criteria like the Amsterdam criteria. During these years of *PMS2* testing, we have identified and classified a substantial number of *PMS2* variants. The aim of this study was dual. Firstly, we wanted to use the newest guidelines for classification of gene variants to systematically re-evaluate all identified *PMS2* variants in Norway. In addition, we wanted to describe the frequency, spectrum and penetrance of cancers in our cohort of carriers of pathogenic and likely pathogenic *PMS2* variants.

## Materials and methods

### Genetic testing of *PMS2* in Norway

Genetic testing of *PMS2* has been offered to patients at the Medical Genetics Departments at St Olavs University Hospital (StO) in Trondheim, Oslo University Hospital (OUS), Haukeland University Hospital in Bergen (HUS) and University Hospital of Northern Norway in Tromsø (UNN) since 2006. The inclusion criteria have varied over the years: Initially we used the Amsterdam and Bethesda criteria, and tested only the MMR-genes in individuals who fulfilled the criteria and/or families with MSI-high tumours or lack of staining of PMS2 on IHC. As access to testing has improved, criteria for genetic testing has become less strict. Testing is now offered to families with only one case of CRC below 60 years. However, testing criteria have not changed at the same time at each hospital, and are not the same for each hospital today. At HUS, testing of *PMS2* is only offered in families where there have been cases of CRC, whereas at the other hospitals, *PMS2* is included in panels that are offered also in families where there have not been cases of CRC.

### Sequencing analyses of DNA and RNA

For mutation detection of the *PMS2* gene, DNA was extracted from EDTA-blood samples using standard protocols. All coding exons of *PMS2* with flanking introns were amplified and sequenced either by Sanger or Next Generation Sequencing (NGS; pyro sequencing with GS Junior, Roche 454 or probe enriched gene panel testing with MiSeq or NextSeq from Illumina). Primers used for Sanger and pyro sequencing were checked for the presence of SNPs under primers and designed to avoid amplification of highly homologous sequences from pseudogenes. All variants detected by NGS were verified with Sanger sequencing.

Long Range-PCR or RNA-analyses (sequencing of cDNA) were performed to verify that a variant belonged to the *PMS2* gene and not to the pseudogenes [8]. cDNA analyses of *PMS2* were also performed in cases of putative splice effects, or when IHC results indicated absence of PMS2 protein expression, and no sequence variants with impact on disease were detected. RNA was isolated from lymphocyte cells (Heparin blood) grown for 4 days in a medium containing Puromycin to prevent Non-sense mediated mRNA decay. Primer and sequence details for both DNA and RNA are available on request.

### Multiplex ligation-dependent probe amplification

For detection of large deletions or duplications (copy number variation; CNV) within *PMS2*, MLPA (SALSA MLPA Kit P008 from MRC-Holland, Amsterdam, Netherland) was used according to the manufacturer’s recommendation. The PCR fragments were separated by capillary electrophoresis, and the results analysed by use of the software Coffalyser (MRC-Holland). In samples with deletion in only one marker (probe site), the corresponding exon were sequenced to exclude the absence of a single nucleotide variant that could cause a false CNV result. Duplications were verified with RNA-analysis when samples were available.

### Microsatellite (MSI) and immunohistochemistry (IHC) analysis

MSI and IHC analyses were performed on formalin-fixed paraffin-embedded material as previously described [9]. MSI was performed using standard multiplex PCR and fragment separation by capillary electrophoresis. If more or fewer than 30% of the repeats were unstable, the tumour was classified as MSI-high (MSI-H) or MSI-low, respectively. Tumours with no shift in number of repeats compared to normal tissue were classified as microsatellite stable. IHC was performed by incubating tissue sections with monoclonal antibodies against PMS2 proteins, and thereafter examination of expression of PMS2 proteins in the nucleus and adjacent non-neoplastic tissue elements and subsequently defined as showing presence or absence of the PMS2 protein.

### Collection and re-evaluation of variants

For this study, we collected and re-evaluated all class 2–5 variants that had been identified in the diagnostic genetic laboratories at StO, OUS, HUS and UNN since 2006 and until 2021. Reference sequence used for the *PMS2* gene was NM_000535.5 and the nomenclature of variants is as recommended by the Human Genome Mutation Committee [10] *PMS2* variants were classified into five classes according to the guidelines provided by The American.

College of Medical Genetics and Genomics (ACMG) from 2015 [11] and CanVIG-UK [12]. We have also used the MMR gene specific guidelines from The International Society for Gastrointestinal Hereditary Tumours (InSiGHT; https://www.insight-group.org/). The five classes are (5) pathogenic, (4) likely pathogenic, (3) variant of uncertain significance (VUS), (2) likely benign, or (1) benign. Alamut Visual version 2.11 (Interactive Biosoftware, Rouen, France) and literature search were used for the classification. We looked for whether the variants had a ClinVar entry with classification (https://www.ncbi.nlm.nih.gov/clinvar/) or whether they were reported to the InSIGHT database (https://databases.lovd.nl/shared/genes/PMS2), and we included frequency data from gnomADv2.1.1 (http://gnomad.broadinstitute.org/) [13] which to date comprise data from almost 250,000 alleles.

### Collection of clinical information

From the electronic patient records at the StO, HUS, OUS and UNN, we collected the following information for all carries of class 4 or 5 variants: Variant, gender, age of positive test result, cancer diagnosis, age of cancer diagnosis and age at last follow-up. There was incomplete information on prophylactic hysterectomy and oophorectomy in female carriers.

### Statistics

We used descriptive statistical methods to calculate frequency of different cancer in carriers, and the Kaplan-Meier algorithm was used to calculate cumulative risk of any cancer, CRC and endometrial cancer. The analyses were performed for men and women separately and together. Risk was calculated from birth. Carriers were scored as affected at time of diagnosis of the cancer in question, or censored as unaffected at last observation or at death if dead for another reason.The Log-rank test was used to compare cumulative risk of cancer in carriers of the founder variant c.989-1G > T and carriers of other *PMS2* variants, and the Chi-square test was used to compare prevalence of cancer at 40, 50, 60 and 70 years in these two cohorts. One homozygous carrier was excluded from the statistical analyses.

All analyses were conducted using SPSS version 28.

### Ethics

The study was approved by the Regional Ethics Committee (Application number 30374). All genetic testing was done according to Norwegian legislation. The legislation requires genetic counselling before predictive genetic testing and an informed consent from the patients.

## Results

### Classification of *PMS2* variants

Data from genetic testing of the *PMS2* gene in Norway for 15 years included around 5000 patients. In total, 220 different *PMS2* variants were detected, and the number of variants in different classes are shown in Table 1. Some of the variants had old classifications and those were re-evaluated. No class 4–5 variants were reclassified. However, many class 3 variants were reclassified to likely benign/ benign because new information about frequency and functional effect were available.

**Table 1:** Classification of the 220 identified variants in PMS2

Class	Number (%)	Variants in
4–5	29 (13.2%)	Table 2
3 (VUS)	48 (21.8%)	Supplementary table S1
1–2	143 (65%)	Supplementary table S1

Twenty-seven of the 29 class 4–5 variants had a ClinVar entry with classification, and 24 of these had similar classification as in the present study. Nineteen of the class 4–5 variants were reported and classified in the InSIGHT (LOVD) database, whereof seventeen had the same class (Table 2). For three and two variant respectively, there was some discordance between our updated classification and the ClinVar and InSIGHT databases. These variants were c.130G > C, c.137G > A, c.(1144 + 1_1145-1)_(2174 + 1_2175-1)dup and c.2113G > A.

**Table 2:** Pathogenic (class 5) and likely pathogenic (class 4) *PMS2* variants identified in our cohort

DNA variant	cDNA protein level	dbSNP rs number	ClinVar# / LOVD, Insight	Class in present study	Number of carriers (number of families)
c.23 + 1G > A	p.(?)	587,782,074	LP, P / NR	LP	1 (1)
c.130G > C	p.(Glu44Gln)	786,202,669	VUS / NR	LP	16 (4)
c.137G > A	p.(Ser46Asn)	121,434,629	LP, P / VUS*	LP	¤
c.537 + 1G > T	r.[=], [354_537del, 354_586del, 354_589del] p.(Ser118Argfs*22), p.(Asp119Argfs*52) p.(Ser118Argfs*52)	863,224,450	NR / NR c.537 + 1G > C c.537 + 1G > A LP	P	6 (2)
c.598del	p.(Val200*)	no	P / NA	P	24 (7)
c.631 C > T	p.(Arg211*)	760,228,510	P / P, VUS*	P	14 (5)
c.736_741delins11	p.(Pro246Cysfs*3)	267,608,150	P / P*	P	3 (1)
c.803 + 1_804-1)_(903 + 1_904-1)del	p.(Tyr268*) Del ex8	no	P / P*	P	9 (2)
c.823 C > T	p.(Gln275*)	587,780,062	P / P	P	27 (3)
c.861_864del	p.(Arg287Serfs*19)	267,608,154	P / P*	P	3 (2)
c.989-1G > T	r.[=, 989_1144del, 989_1015del] p.(Glu330_Glu381del; Glu330_Pro338del)	587,780,064	P / P*	P	204 (58)
c.1112_1113delinsTTTA	p.(Asn371Ilefs*2)	587,779,326	P / P*	P	1 (1)
c.1239dup	p.(Asp414Argfs*44)	758,048,239	P / P	P	1 (1)
c.1261 C > T	p.(Arg421*)	587,778,617	P / LP, P*	P	1 (1)
c.1345 C > T	r.[=, 1345c > t] p.(Gln449*)	876,661,256	P / NR	P	1 (1)
c.(1144 + 1_1145-1)_(2174 + 1_2175-1)dup	r.[=, 1145_2174dup]/ p.(Pro726*) Dup ex11-12	no	LP / P*	P	34 (12)
c.1738 A > T	p.(Lys580*)	267,608,169	P / P*	P	1 (1)
c.1831dupA	p.(Ile611Asnfs*2)	63,750,250	P / P*	P	3 (2)
c.1882 C > T	p.(Arg628*)	63,750,451	P / VUS, P*	P	1 (1)
c.1939 A > T	p.(Lys647*)	201,451,115	P / P*	P	1 (1)
c.1970delA	p.(Asn657Ilefs*8)	1,064,794,566	LP, P / NR	P	19 (3)
c.2041 C > T	p.(Gln681*)	1,782,465,728	P / NR	P	¤
c.2113G > A§	p.(Glu705Lys)	267,608,161	VUS, LP, P / P, VUS*	LP	45 (10)
c.2156delA	p.(Gln719Argfs*6)	786,201,062	P / P	P	15 (5)
c.(2174 + 1_2175-1)_(2445 + 1_2446-1)del	p.(Pro726*) Del ex13-14	No	NR / NR	P	1 (1)
c.2192_2196del	p.(Leu731Cysfs*)	63,750,695	P / P*	P	4 (1)
c.2382dupT	p.(Gly795Trpfs*29)	1,231,406,078	P / NR	P	8 (2)
c.2404 C > T	p.(Arg802*)	63,751,466	P / P*	P	1 (1)
c.2413 C > T	p.(Gln805*)	1,554,293,810	P / NA	P	¤

# VUS: variant of uncertain significance, LP: Likely pathogenic, P: Pathogenic

*Classified by the InSIGHT group

¤ Clinical data missing

NR: not reported

NA: In LOVD, not classified

The *PMS2* missense variant c.130G > C, p.(Glu44Gln) has been reported as a class 3 variant in ClinVar and was not reported to InSIGHT. We have interpreted it as likely pathogenic based on several criteria. It has been found in CRC patients, and not in control populations (ExAC, gnomADv2.1.1, 1000 genomes, dbSNP), and ACMG criteria PM2_supportive can therefore be used. The four reports in ClinVar were based on clinical testing for LS/CRC. We have detected the variant in four families (later also in three more families). In these families, five carriers have all been diagnosed with CRC, four of these diagnosed in their 40- and 50ies. The fifth is a homozygous carrier who was diagnosed with CRC the first time at 21 years old and later with several new primary colorectal tumours. Thus, he has a phenotype of constitutional MMR deficiency (CMMRD). Tumour screening analyses showed MSI-H in 5/7 tumours in carriers of this variant (ACMG criteria PP4_strong), while IHC showed normal staining for PMS2 protein in 7/7 tumours. All in silico prediction programs interpret *PMS2* c.130G > C to be pathogenic (REVEL score 0.872; ACMG criteria PP3), probably because the Glutamic acid in codon 44 is highly conserved and it is located next to the Asparagine in codon 45, which is both an ATP and a Mg2 + binding site. The combined evidences (ACMG criteria) give a class four variant.

The c.137G > A, p.(Ser46Asn) was classified as a VUS in the InSIGHT database, while it is reported as likely pathogenic and pathogenic in ClinVar and the present study classify it as a likely pathogenic variant. This discordance may be because the InSIGHT classification has not been updated since 2013. It is interpreted to be pathogenic (REVEL score 0.811), and it has been shown to give reduced repair activity [14]. Studies have identified the variant homozygote or compound heterozygote in CMMRD patients [15–17]. It has low frequency in gnomAD, and another variant in the same codon (c.137G > T) has been reported as pathogenic both in ClinVar and in InSIGHT.

The c.(1144 + 1_1145-1)_(2174 + 1_2175-1)dup variant (duplication of exon 11 and 12) has been interpreted as likely pathogenic (class 4) in ClinVar. We have performed cDNA analysis of samples from five carriers which showed that the duplication of exon 11–12 was in tandem This is interpreted to lead to replacement of Proline in codon 726 with a stop codon. Tumours from two carriers of this variant were MSI-H and lacked PMS2 expression on IHC. Together these evidences make up a class 5 pathogenic variant.

The missense variant c.2113G > A, p. (Glu705Lys) has been reported as VUS (class 3), likely pathogenic (class 4) and pathogenic (class 5) in ClinVar, while in InSIGHT it is classified as VUS and pathogenic. The highly conserved amino acid in codon 705 is in the metal binding motif, and the substitution from Glutamic acid (acidic) to Lysine (basic), changes the charge from negative to positive polarity. We have interpreted the variant to be likely pathogenic based on functional studies showing that the variant disrupts protein function [14, 18–20]. In addition, 5/6 tumours in carriers were MSI-high and 3/7 tumours lacked expression of PMS2 on IHC.

Two of the pathogenic variants (Table 2) had no ClinVar reports and no frequency in gnomADv2.1.1. The c.537 + 1G > T variant became a class 5 variant out from cDNA analysis which showed aberrant splicing leading to three alternative transcripts: one missing exon five and two missing exon five and parts of exon six. Two other variants in the same nucleotide (c.537 + 1G > C and c.537 + 1G > A) have been interpreted as likely pathogenic (class 4) in ClinVar.

The c.(2174 + 1_2175-1)_(2445 + 1_2446-1)del variant (deletion of exon 13 and 14) is interpreted to lead to a new stop codon, and it is therefore classified as pathogenic by us. This variant is not reported to ClinVar. However, other similar variants (deletion of one or more exons in the 5’end of PMS2) are reported as pathogenic in ClinVar.

Altogether 444 individuals from 124 families harboured one of the 29 class 4 and 5 variants (Table 2). The most common class 4/5 variant was the founder variant c.989-1G > T that originates from Mid-Norway. This variant was found in 204 individuals and 58 families, comprising 46.4% (204/440) of all carriers and 46.8% (58/124) of all families. Thirteen of the class 4/5 variants (13/29 = 44.8%) were only found in one family.

The 48 class 3 variants included 39 missense, one silent, four intronic, one 5’UTR, one 3’UTR, one whole gene duplication and one in frame deletion (Supplementary table S1). Out of these 48 VUSes, 34 had a classification in accordance with ClinVar, and for one VUS (c.-50G > T) our classification was dissimilar to ClinVar. We chose to keep this 5’UTR variant as a VUS, because it alter a G belonging to a CpG island with binding site for H3K4Me and H3K27Ac. Twelve VUSes had no report in ClinVar. The missense variant c.2249G > A p.(Gly750Asp), which we had interpreted as a VUS with potential to be pathogenic, had several reports in ClinVar and most of them as likely pathogenic. When we re-evaluated this variant, we agree that it is likely pathogenic because it has been found as compound heterozygous in several patients with a Constitutional mismatch repair deficiency syndrome phenotype [21, 22]. However, it may be a hypermorphic variant, because it displayed repair efficiencies higher than a pathogenic variant, but lower compared to wild type in one functional study. The author suggested that the variant might be pathogenic with reduced penetrance [14].

Out of the 143 class 1–2 variants in our study, 104 were in accordance with ClinVar reports. Nine variants were classified as a VUS in ClinVar, while we had interpreted these 9 missense variants as class 2. Thirty class 1–2 in our study had no reports in ClinVar.

### Prevalence of cancer in carriers of pathogenic variants and cumulative risk

In total, 444 heterozygous carriers and 38 obligate carriers of class 4 or 5 *PMS2* variants were identified. One hundred and sixty-six of these (166/482 = 34%) had been diagnosed with any kind of cancer. Mean age was 54.5 (range 21–82) years. Average age at last follow-up was 49 years.

CRC was the most common cancer in both men and women. Eighty-seven carriers (87/482 = 18%) had been diagnosed with one or more CRC. Mean age of diagnosis of the first CRC tumour was 54.0 (range 24–86). Two of these patients (2/87 = 2.3%) were diagnosed with CRC before the age of 30, 10 (10/87 = 11.5%) before the age of 40, and 34 (34/87 = 39%) before the age of 50 (Table 3). No carriers had synchronous CRC, but five were diagnosed with two metachronous CRC tumours, and one patient with three tumours. Eight patients had more than one LS associated tumour.

**Table 3:** Frequency of colorectal cancer in different age cohorts

Age cohort	*PMS2* carriers (*n* = 87)
> 30	2 (2.3%)
30–39	8 (9.2%)
40–49	24 (27.5%)
50–59	23 (26.4%)
60–69	18 (20.7%)
70-	12 (13.8%)

Thirty-two women (32/262 = 12.2%) had been diagnosed with endometrial cancer. Mean age of diagnosis was 55.4 years (range 21–86).

Breast cancer was the third most common cancer in women after CRC and endometrial cancer affecting 17/262 (6.5%), mean age 55.4 (range 39–81).

Other kinds of cancer were also seen in carriers: ovarian cancer, prostate cancer, lung cancer, pancreatic cancer, melanoma, and gastric cancer (Table 4).

**Table 4:** Frequency of cancer in male and female *PMS2* carriers

Cancer	All (*n* = 482)	Women (*n* = 262)	Men (*n* = 220)
Colorectum	87 (18.0%)	36 (13.7%)	51 (23.8%)
Endometrium	-	32 (12.2%)	
Ovary	-	5 (1.9%)	
Prostate	-		8 (3.6%)
Urinary tract	1 (0.2%)	1 (0.4%)	-
Breast	-	17 (6.5%)	

The estimated cumulative CRC risk in the total cohort of carriers was 34.2% at 70 years, 25.4% for women and 43.9% for men. Cumulative risk for endometrial cancer was 26.1% at age 70 years. For the c.989-1G > T carriers, the cumulative CRC risk at 70 years was 37.5% and for carriers of the other variants, 31.8% (Table 5). The observed difference in CRC risk between these two cohorts of carriers was not statistically significant according to the Log-rank test.

**Table 5:** Cumulative risks of cancer in *PMS2* carriers

	Cumulative risk (standard error)			
	40 yrs	50 yrs	60 yrs	70 yrs
**All carriers**: Any cancer	4.5% (0.010)	18.9% (0.022)	36.7% (0.030)	57.9% (0.036)
CRC, all carriers	3.1% (0.009)	10.8% (0.017)	21.7% (0.026)	34.2% (0.036)
CRC, women	2.9% (0.012)	7.6% (0.020)	16.5% (0.031)	25.4% (0.045)
CRC, men	3.3% (0.013)	14.6% (0.029)	27.8% (0.041)	43.9% (0.054)
Endometrial cancer	1.4% (0.008)	7.3% (0.021)	18.3% (0.036)	26.1% (0.046)
**c.989-1G > T carriers**: Any cancer	5.3% (0.017)	17.9% (0.032)	37.5% (0.047)	62.8% (0.056)
CRC, all carriers	3.6% (0.015)	10.5% (0.026)	21.6% (0.041)	37.5% (0.058)
CRC, women	4.5% (0.022)	7.3% (0.029)	16.8% 0.049)	30.0% (0.074)
CRC, men	3.9% (0.022)	14.5% (0.045)	27.8% (0.068)	46.5% (0.088)
Endometrial cancer	2.2% (0.015)	6.4% (0.028)	21.6% (0.058)	26.5% (0.072)
**c.989-1G > T carriers excluded**: Any cancer	4% (0.013)	19.4% (0.029)	36.1% (0.038)	54.5% 0.047)
CRC, all carriers	2.7% (0.011)	11.0% (0.023)	21.9% (0.033)	31.8% (0.044)
CRC, women	1.7% (0.012)	7.9% (0.027)	16.4% (0.041)	21.9% (0.054)
CRC, men	3.9% (0.019)	14.7% (0.038)	27.9% (0.052)	42.0% (0.068)
Endometrial cancer	1.7% (0.012)	8.0% (0.030)	16.0% (0.044)	26.3% (0.062)

## Discussion

In this national study of *PMS2* mutation screening performed since 2006, we identified a substantial number of gene variants. Most of the variants with a ClinVar report had similar classes as in our study. However, we disagree with a few of them, and 44 variants had no ClinVar report (two class 4/5 and 42 class 1–3 variants). We have performed cDNA analyses for many of the variants, supporting the classification of them. In example, two variants with no report in ClinVar could be evaluated to class 5 variants, because of cDNA analyses (Table 2).

In the Norwegian population, we have identified 29 different class 4/5 variants. The founder mutation c.989-1G > T was the most common variant and was found in 46.7% of all carriers. Almost half of all other class 4/5 variants were found in only one family each. CRC was the most commonly observed cancer in both men and women, affecting 25.4% of women and 43.9% of men by the age of 70.

Several previous studies have demonstrated that risk of CRC is only moderately increased in *PMS2* carriers. Ten Broeke and colleagues did a retrospective study that included 513 confirmed carriers and their first and second-degree relatives. They found a cumulative risk of CRC at 80 years was 12% for women and 13% for men [4]. Similarly, the International Mismatch Repair Consortium found that the majority of carriers had a cumulative risk of CRC that was less than 20% [23]. Penetrance of CRC in our study cohort was 25.4% and 43.9% for women and men respectively. This is similar to what we have previously reported for the *PMS2* c.989-1G > T variant only [24]. Almost 50% of carriers in the current study had this variant, but we did not find a significant difference in cumulative risk of CRC or endometrial cancer between carriers of this variant and carriers of other *PMS2* variants. However, both these studies are based on retrospective data from all identified carriers, including affected probands. Even though testing the last years has been offered also to families with limited number of CRCs and not only to those fulfilling the Amsterdam or Bethesda criteria, our estimates may be affected by selection bias and therefore higher than the true risk of CRC in healthy *PMS2* carriers. Moreover the inclusion criteria for testing have varied over the years and between the hospitals. We therefore cannot delineate exactly to what extent our estimates are affected. This is a clear limitation to our study. Prospective studies of risk in general provide more reliable estimates of risk. In their study from 2020, the prospective LS database included 6350 MMR carriers, of which only 407 carried a pathogenic variant in *PMS2.* They found that *PMS2* carriers did not have an increased CRC risk below 50 years, and that the risk was not significantly increased after 50 years [2]. In contrast to their observations, we found that 11.5% of *PMS2* carriers with CRC were diagnosed before 40 years of age. Similar findings have been reported by others, indicating that the penetrance of pathogenic *PMS2* variants is variable [4, 25]. The International Mismatch Repair Consortium found an increased Hazard Ratio (HR) for CRC in *PMS2* carriers below 40 years. HR varied between male and female carriers and between the continents, but in male carriers from Australasia it was as high as 26.8 [23].

Because cancer risk is different for the different MMR genes, there is an ongoing discussion on whether all carriers should be recommended the same surveillance. For *PMS2* carriers, some have suggested that start of colonoscopic surveillance could be delayed from 25 to 35 years [4, 5] and that female carriers should not be recommended prophylactic hysterectomy [4, 26]. In Norway, *PMS2* carriers are informed of their moderate CRC risk, but all MMR carriers are currently recommended biannual colonoscopies from the age of 25. Based on the reported varying penetrance of CRC in *PMS2* carriers, it may be too early to draw any firm conclusions regarding surveillance. At the time of data collection, our cohort included 444 carriers of class 4/5 variants in *PMS2*. Our national cohort now includes around 600 carriers. This large sample size combined with the high quality data on cancer diagnoses that is available from the Cancer Registry of Norway may enable us to explore risk of colorectal and other cancers in different age groups of carriers further in a prospective study, and this may give more reliable estimates of risk.

Breast cancer was the third most common cancer in female carriers, affecting 6.5%. Whether or not there is an increased risk of breast cancer in MMR carriers has been investigated in several studies, providing conflicting results [2, 27]. Cumulative risk of breast cancer in our cohort was 16.4%, compared to 10.7% by 80 years in the general population in Norway [28]. We cannot exclude that risk of breast cancer may be slightly increased, but breast cancer is the most common cancer in women and the observed difference may be due to chance, or ascertainment bias.

## Conclusions

At the time of data collection, 29 different class 4/5 variants had been identified in the Norwegian population. The founder mutation c.989-1G > T was the most commonly observed variant and was found in almost half of all carriers. Penetrance of colorectal cancer by 70 years was 34.2% for all carriers, 43.9% for males and 25.4% for women. Our retrospective estimates may be affected by selection bias, should be interpreted with caution and confirmed in prospective cohorts. However, the observation that 11.5% of those who had been diagnosed with CRC were younger than 40 years at time of diagnosis, confirms the variable penetrance of *PMS2.*

## Electronic supplementary material

Below is the link to the electronic supplementary material.

Supplementary Material 1

## Data Availability

Data that support the findings of this study are available upon request from the corresponding author. The data file with clinical data is not available for sharing because of sensitive data that could lead to compromising of individual privacy.

## Abbreviations

ACMG: The American College of Medical Genetics and Genomics
CMMRD: Constitutional MMR deficiency
CNV: Copy number variation
CRC: Colorectal cancer
EC: Endometrial cancer
HUS: Haukeland University Hospital
IHC: Immunohistochemistry
LS: Lynch syndrome
MLPA: Multiplex Ligation-dependent Probe Amplification
MMR: Mismatch repair
MSI: Microsatellite instability
MSI-H: MSI-high
NGS: Next Generation Sequencing
OUS: Oslo University Hospital
StO: St Olavs University Hospital
UNN: University Hospital of Northern Norway
VUS: Variant of uncertain significance
