# Supplementary material for: PMS2 mutation spectra in Norway and risk of cancer for carriers of pathogenic variants
Source: Hered Cancer Clin Pract. 2024 Sep 27;22:20. doi: 10.1186/s13053-024-00292-6 (PMC11438158; doi:10.1186/s13053-024-00292-6)
Supplement: Supplementary file 1 — Supplementary Material 1 [file 13053_2024_292_MOESM1_ESM.pdf]

Sjursen et al, Hereditary Cancer in Clinical Practice

Supplementary table: Class 1-3 *PMS2* variants identified in Norway.

Variant (NM_000535.5)	dbSNP rs number	gnomAD frequency (%) ¤	ClinVar	LOVD InSight*	Class in this study	Comments
c.(?_-87)_(*160_?)dup p.(?)			-	-	3	Whole gene duplication
c.-195T>C	2302336	6.187	B	VUS B*	1	
c.-154C>G	3735296	16.50	B	VUS, B*	1	
c.-93G>T	6976537	0.76	B	B	1	
c.-93G>C	6976537	0.32	B	B	1	
c.-53G>A	559634583	0.16	B	VUS, B	1	
c.-50G>T	557908122	-	LB	-	3	In one patient. Nucleotide not conserved. Alter a G belonging to a CpG island with binding site for H3K4Me3 and H3K27Ac
c.18C>G p.(Ser6Arg)	557908122	-	VUS	-	2	Neither nucleotide nor amino acid are Conserved. Predicted tolerated.
c.23+10G>C	192027828	0.18	B	LB, B	1	cDNA: Do not affect splicing
c.23+72C>T	3735295	23.18	B	VUS, B	1	
c.52A>G p.Ile18Val	63750123	0.91	LB, B	VUS, LB*, B	1	cDNA: Do not affect splicing

c.59G>A p.Arg20Gln	10254120	7.27	B	VUS, LB, B*	1	
c.82T>C p.(Ser28Pro)	587781908	0.0012	VUS	NA	3	Amino acid highly conserved. Predicted deleterious.
c.86G>C p.(Gly29Ala)	146176004	0.059	VUS, LB, B	VUS, LB	1	1 homozygote in gnomADv2.1.1 0,6% Ash Jewish
c.119A>G p.(Lys40Arg)	-	-	-	NA	3	Normal IHC/MSS cDNA: Do not affect splicing, (c.A>C VUS ClinVar)
c.124T>A p.(Leu42Ile)	35953120	-	-	-	3	Co-occurrence with pathogenic *POLE* variant, MSS/norm IHC
c.163+10G>A	746034469	-	-	-	2	No interpretation of aberrant splicing Nucleotide not conserved
c.166C>G p.(Leu56Val)	371011390	0.0047	LB, B	VUS, LB	2	
c.180C>G p.Asp60Glu	200313585	0.098	B, LB	VUS*, LB, B	2	
c.197T>C p.(Ile66Thr)	769554577	0.0021	VUS	VUS	3	
c.207A>G p.(Ser69=)	1583410659	-	LB	-	2	cDNA: Do not affect splicing
c.211A>G p.(Asn71Asp)	1562695328	-	-	NA	3	
c.240C>T p.(Phe80=)	143162541	0.0067	LB, B	LB	2	
c.250+24A>G	370191165	0.038	-	VUS	1	
c.251-72A>G	117831773	1.96	B	VUS, B*	1	
c.251-20T>G	149343081	0.083	B	VUS, LB	1	cDNA: Do not affect splicing
c.255G>A p.(Leu85=)	200491279	0.018	B, LB	VUS, LB	2	Partly mis-splicing of exon 4 (<20 % mis-splicing), Do not segregate with disease
c.286G>A p.(Ala96Thr)	772740220	-	VUS	-	3	

c.288C>T p.(Ala96=)	12532895	7.70	B	VUS, B*	1	
c.348A>C p.(=)	763057312	0.0030	LB, VUS	-	2	
c.354-34C>G p.(?)	200029834	0.05	LB/ VUS	VUS, LB	2	No interpretation of aberrant splicing<br>Nucleotide not conserved
c.372C>G p.(Thr124=)	1060504836	-	LB	-	2	Found together with PMS2 c.537+1 G>T<br>No interpretation of aberrant splicing
c.378 C>T p.(His126=)	768488890	0.0020	LB	-	2	cDNA: Do not affect splicing
c.379G>A p.(Ala127Thr)	114090343	0.044	B, LB, VUS	LB	2	
c.383 C>T, p.(Ser128Leu)	116373169	0.068	B, LB	VUS, LB	1	cDNA: Do not affect splicing
c.403C>G p.(Leu135Val)	876658423	0.00041	VUS	NA	3	
c.433C>A p.(Gln145Lys)	786204133	0.0032	VUS, LB	NA	3	
c.434A>G p.(Gln145Arg)	1064795040	-	VUS	-	3	
c.477G>A p.(Val159=)	147701251	0.0078	B, LB	LB	2	
c.564A>C p.(Leu188Phe)	-	-	-	-	3	
c.620G>A p.(Gly207Glu)	374704824	0.035	VUS, LB	VUS*	2	
c.630A>G p.(Lys210=)	765847615	0.0012	LB, B	LB	2	Co-occurrence with BRCA1 pathogenic variant
c.632G>A p.(Arg211Gln)	587781934	0.0042	VUS X10	VUS	2	
c.705+17A>G	62456182	36.52	B	VUS, B*	1	
c.705+36C>T	111908557	1.28	B	B	1	
c.706-5_706-4del	776641246	8.98	B, LB	B	1	

p.(?)						
c.706-4dup<br>p.(?)	7745388<br>69	7.31	B	LB, B	1	
c.708G>T<br>p.(Leu236Phe)	2013956<br>30	0.0044	VUS, LB	LB	3	
c.751G>A<br>p.(Val251Met)	1424340<br>11	0.0022	VUS, LB	VUS	3	
c.780C>A<br>p.(Ser260=)	1805319	0.00041	LB, B	LB	2	
c.780C>G<br>p.(Ser260=)	1805319	80.36	B	VUS, B*	1	
c.791A>G<br>p.(His264Arg)	1060503<br>129	0.00071	VUS	-	3	
c.795T>C<br>p.(Asn265=)	7666671<br>86	0.0018	LB	-	2	No interpretation of aberrant splicing
c.831G>A<br>p.(Thr277=)	1164815<br>22	0.0088	LB, B	LB	2	
c.857A>G<br>p.(Asp2866Gly)	1167886<br>08	0.013	VUS, LB	VUS	3	
c.878A>G<br>p.(Asn293Ser)	1274671<br>450	0.0064	VUS	NA	3	
c.895C>T<br>p.(Pro299Ser)	1562663<br>421	-	VUS	-	3	
c. 904-20A>G	3751147<br>86	0.0022	LB	LB	2	cDNA: Normal transcripts
c.930C>T<br>p.(Tyr310=)	1405625<br>567	0	LB	-	2	No interpretation of aberrant splicing
c.988+11T>C	1399696<br>71	0.059	B	-	1	cDNA: Do not affect splicing
c.989-256A>T	7981507<br>5	5.77	-	-	1	
c.989-162G>A	2869947<br>0	34.63	-	-	1	
c.989-105dup	1492497<br>85	16.15	-	-	1	

c.993C>T p.(Cys331=)	1865772 15	0.0029	LB	-	2	
c.1004A>G p.(Asn335Ser)	2005130 14	0.028	B, LB, VUS,	VUS, LB	3	
c.1004A>T p.(Asn335Ile)	2005130 14	0.00041	VUS	VUS	3	Normal IHC/MSI (CRC 60 year) cDNA: Do not affect splicing
c.1057G>A p.(Ala353Thr)	5877828 26	0.0032	VUS	-	3	OuS
c.1096G>C p.(Asp366His)	1417690 57	0.0011	LB, VUS	NA	2	
c.1111A>C p.(Asn371His)	1217473 969	-	VUS	-	3	
c.1125G>C p.(Gln375His)	1060504 838	-	-	-	2	
c.1134G>T p.(Leu378=)	7545768 28	-	LB	-	2	
c.1136A>T p.(Asp379Val)	5360317 11	-	VUS	-	3	MAPP/PP2 Prior P: 0.0049
c.1145-116C>T	1112555 73	1.23	-	-	1	cDNA: Normal transcripts
c.1145-102G>A	5495611 07	0.14	-	-	1	cDNA: Normal transcripts MSS and normal protein staining
c.1145-77G>A	-	-	-	-	2	cDNA: Normal transcripts
c.1145-58C>T	9660192 97	0.0032	-	-	2	cDNA: Normal transcripts
c.1145-57G>A	2005630 64	2.24	-	-	1	
c.1170G>A p.(Ala390=)	7555784 13	0.0043	LB	VUS, LB	2	
c.1211C>G p.(Pro404Arg)	5361118 18	0.0080	VUS, LB	VUS	3	
c.1243G>A, p.(Val415Met)	1383876 87	0.017	B, LB, VUS	VUS, LB	2	1x MSS/IHC normal Weakly conserved a.a
c.1245G>A p.(Val415=)	3707018 00	0.0004	LB	LB	2	

c.1248C>A p.(Ser416=)	780709321	0.0035	B, LB	LB	2	
c.1249A>G p.(Ile417Val)	1060503132	-	VUS	NA	3	Weakly conserved a.a.<br>cDNA: Do not affect splicing
c.1249A>C p.(Ile417Leu)	1060503132	0.00040	VUS	NA	2/3	Weakly conserved a.a.
c.1266G>A p.(Glu422=)	138049175	0.10	B, LB	LB, B	2	
c.1270T>C p.(Phe424Leu)	1562635229	-	VUS	-	3	
c.1280G>A p.(Arg427His)	112902065	0.0060	B, LB	NA	2	Orthologs with Histidin in this position
c.1344A>T p.(Gly448=)	759192470	0.00040	LB	LB	2	
c.1357A>G p.(Met453Val)	587780722	0.00080	LB, VUS	NA	2	
c.1360_1361delinsTC p.(Leu454Ser)	587778615	-	VUS	NA	2/3	Normal IH/MSS,<br>cDNA: Do not affect splicing
c.1372A>C p.(Thr458Pro)	779306532	0.0024	VUS	NA	2/3	
c.1379G>A p.(Gly460)Asp	150201462	0.0025	B, LB, VUS	NA	2	Normal IH/MSS<br>Co-occurrence with MLH1 pathogenic variant
c.1398C>T p.(Gly466=)	752666485	0.0024	LB	-	2	
c.1408C>T p.(Pro470Ser)	1805321	38.60	B	VUS, B*	1	
c.1437C>G p.(His479Gln)	63750685	0.46	VUS, LB, B	VUS*, LB, B	1	
c.1437C>T p.(His479=)	63750685	0.0018	VUS, LB, B	**-**	**2**	cDNA: Normal transcripts
c.1438G>A p.(Gly480Arg)	146848345	0.0028	LB, VUS	NA	3	dbSNP: benign
c.1454C>A p.(Thr485Lys)	1805323	7.73	B	VUS, B*	1	

Følsomhet Intern (gul)

c.1470G>A p.(Val490=)	7862030 76	0.00080	LB	-	2	No interpretation of aberrant splicing
c.1476G>A (p.Lys492=)	7663095 32	0.00080	LB	-	2	
c.1477G>A p.(Asp493Asn)	1486420 64	0.00080	VUS	VUS	3	Normal IHC/MSS
c.1481C>T p.(Ser494Leu)	5877826 02	0.0012	VUS	VUS*, LB	3	
c.1487_1489del p.(His496_Gly49 7delinsArg)	-	-	-	-	3	
c.1488C>T p.(His496=)	1805320	0.77	B	VUS, LB, B*	1	
c.1490G>A p.(Gly497Asp)	1997398 59	0.011	LB, VUS	VUS, LB	2	Normal IHC/MSS<br>cDNA: Normal transcripts
c.1510G>C p.(Glu504Gln)	3685167 68	0.0036	VUS, LB	VUS*, LB	3	
c.1531A>G p.(Thr511Ala)	2228007	2.45	B	VUS, B*	1	
c.1532C>T p.(Thr511Met)	7490281 1	0.98	B	VUS, LB, B*	1	
c.1533G>A p.(Thr511=)	5425203 09	0.0088	LB, B	-	2	Normal IHC/MSS<br>No interpretation of aberrant splicing
c.1556A>G p.(Tyr519Cys)	6375064 9	0.0085	LB, VUS	VUS*, LB	2	
c.1557T>C p.(Tyr519=)	6972869	0.32	B, LB	B	1	
c.1559C>T p.(Ala520Val)	6375130 0	0.0071	LB, VUS	VUS, LB	2	
c.1560G>A p.(Ala520=)	2011678 14	0.033	LB	LB, B	2	
c.1567T>A p.(Ser523Thr)	6375113 2	0.016	LB; VUS	VUS, LB	2	
c.1569C>G p.(Ser523=)	1414587 72	0.62	LB, B	VUS, LB*, B	1	

c.1594C>T p.(His532Tyr)	1783037687	-	VUS	-	2	
c.1599G>A p.(Val533=)	2128727076	-	-	-	3	
c.1609G>A p.(Glu537Lys)	115052399	0.12	B, LB	LB, B	2	
c.1621A>G p.(Lys541Glu)	2228006	-	B	B, VUS	1	
c.1656T>C p.(His552=)	113726095	0.0074	LB	B	2	
c.1677A>G p.(Gly559=)	-	-	-	-	2	
c.1688G>T p.(Arg563Leu)	63750668	0.64	LB, B	P, VUS*, LB, B	1	
c.1703C>G p.(Pro568Arg)	869312801	0.00040	VUS	NA	2	
c.1705A>G p.(Thr569Ala)	762151417	0.00080	VUS	-	2	
c.1711C>A p.(Leu571Ile)	63750055	0.28	B, LB	VUS, LB*, B	1	
c.1717A>T p.(Thr573Ser)	63751211	0.0071	LB, VUS	VUS, LB	2	
c.1753C>A p.(Leu585Ile)	63750947	0.0011	LB, VUS	VUS*, LB	2	Co-occurrence with PMS2 pathogenic variant, not CMMRD
c.1765G>C p.(Asp589His)	749727182	0.00080	VUS, LB	NA	2	
c.1766A>G p.(Asp589Gly)	774767419	-	VUS	NA	2	
c.1774C>G p.(Gln592Glu)	63750994	-	VUS	-	2	
c.1777A>C p.(Lys593Gln)	1212790997	-	VUS	-	3	
c.1789A>T p.(Thr597Ser)	1805318	0.86	B	VUS, LB, B*	1	
c.1828A>G p.(Lys610GLu)	199700509	0.0028	VUS, LB	VUS	3-	Normal IH/MSS<br>cDNA: Normal transcripts

c.1866G>A p.(Met622Ile)	1805324	1.73	B	VUS, LB, B*	1	
c.1872T>G p.(Ser624=)	-	-	-	-	2	
c.1936A>C p.(Arg646=)	369582237	0.0039	LB, VUS	-	2	
c.1956T>G p.(Ile652Met)	267608168	-	-	VUS	3	
c.1978G>A p.(Ala660Thr)	2128720257	-	VUS	-	3	
c.1981G>A p.(Glu661Lys)	778531080	0.0016	VUS	VUS	2	Normal IHC/MSI
c.1992A>G p.(Leu664=)	-	-	-	-	2	
c.2006+6G>A	111905775	7.37	B	VUS, B*	1	
c.2007-411A>G	55782426	40.22	-	-	1	
c.2007-339G>A	4724765	49.18	-	-	1	
c.2007-287A>G	2692555	21.58	-	-	1	
c.2007-7C>T	55954143	8.38	B	VUS, B*	1	
c.2007-6C>G	376018314	0.0027	B, LB, VUS	B	3-	
c.2007-4G>A	1805326	15.54	B	VUS, B*	1	
c.2012C>T p.(Thr671Met)	587780046	0.018	B, VUS	LB	2	
c.2013G>A p.(Thr671=)	771513870	0.0012	LB	-	2	
c.2068A>C p.(Lys690Gln)	587781909	0.0044	VUS	VUS	3	

c.2086T>C p.(Phe696Leu)	-	-	VUS	-	3	
c.2089A>G p.(Ile697Val)	1554295 859	-	VUS	-	3	
c.2108C>T p.(Thr703Met)	3701967 22	0.0075	VUS, LB	VUS	2	cDNA: Normal transcripts, Co-occurrence with PMS2 pathogenic variant, not CMMRD
c.2127C>T p.(Phe709=)	1999437 48	0.0060	B, LB, VUS	LB	2	
c.2149G>A p.(Val717Met)	2016713 25	0.071	B, LB, VUS	VUS, LB	1	Co-occurrence with MLH1 pathogenic variant
c.2160G>A p.(Gly720=)	5464410 38	0.050	LB, B	-	2	
c.2174+6T>C	5877800 50	0.0040	VUS	-	3-	cDNA: Normal transcripts
c.2174+64G>A	2128701 528	-	-	-	3	
c.2187C>G p.(Leu729=)	3736305 35	0.094	B, LB	VUS, LB, B	1	cDNA: Normal transcripts
c.2247T>C p.(Asn749=)	2008248 31	-	LB	-	2	
c.2249G>A p.(Gly750Asp)	5877793 37	0.00040	VUSx2, LPx7, Px1	P, VUS*	3+	
c.2253T>C p.(Phe751=)	1805325	0.021	B	VUS, B*	1	
c.2266G>A p.(Asp756Asn)	7622063 30	0.0039	VUS	-	3	
c.2275+25C>T	1841151 26	0.33	B	-	1	
c.2275+42T>C	1813802 55	0.24	B	LB	1	cDNA: Normal transcripts
c.2275+117G>C	2429573	0.33	-	-	1	cDNA: Normal transcripts
c.2275+126G>C	1472510 24	0.83	-	B	2	
c.2275+130A>G	-	-	-	-	2	cDNA: Normal transcripts

c.2275+164C>T	1221581593	0.026	-	-	2	cDNA: Normal transcripts
c.2275+169G>C	12537416	6.99	-	-	1	
c.2275+169del	201067462	1.34	-	-	1	
c.2275+234G>C	559629761	0.18	-	-	1	cDNA: Normal transcripts
c.2276-135T>C	9655490	21.42	-	-	1	
c.2276-115G>T	1349834311	-	-	-	3-	cDNA: Normal transcripts
c.2324A>G p.(Asn775Ser)	17420802	0.033	B	VUS, LB, B*	1	Homozygotic in one patient
c.2340C>T p.(Pro780=)	142230276	0.041	B	VUS, LB, B*	1	Homozygotic in one patient
c.2350G>A p.(Asp784Asn)	143340522	0.12	B, LB, VUS	VUS, LB	2	
c.2356C>A p.(Leu786Met)	576055272	0.10	LB, B, VUS	LB	2	OuS
c.2380C>T p.(Pro794Ser)	773393960	O.00081	VUS	LB	3-	Pseudogene variant?
c.2395C>T p.(Arg799Trp)	149202766	0.050	LB, VUS	LP, VUS*	2	Pseudogene variant?
c.2445+30A>G	549704870	0.63	B	LB, B	1	cDNA: Normal transcripts
c.2445+96C>A	541335663	0.19	-	LB	2	cDNA: Normal transcripts
c.2445+97C>T	574243763	0.013	-	-	2	cDNA: Normal transcripts
c.2445+135C>T	562356357	1.43	-	-	1	cDNA: Normal transcripts
c.2453T>C p.(Ile818Thr)	786202876	0.0016	VUS	-	3	Normal IHC/MSS
c.2466T>C, p.(Leu822=)	10000	11.06	B	VUS, B*	1	
c.2570G>C p.(Gly857Ala)	1802683	28.89	B	VUS, B	1	

Følsomhet Intern (gul)

c.2583G>A p.(Gln861=)	-	-	-	-	2	
c.*16G>C	1218721576	-	-	-	3-	
c.*17G>C	56089649	0.0093	B, LB	VUS, LB, B*	1	
c.*92dupA	267608145	-	B	VUS, B*	1	

¤ Total allele frequency (%) for all populations reported in gnomAD v2.1.1.

VUS: variant of uncertain significance, LP: Likely pathogenic, P: Pathogenic

*Classified by the InSIGHT group.

NR: not reported.

NA: In LOVD, not classified
